# Colour Blindness

**Published:** 1890-03-08

**Authors:** 


### COLOUR BLINDNESS.

Dr. König has recently met with two cases of acquired monochromatism, in which the patients, having previously enjoyed full perception of colour, were able to aver credibly that all objects appeared black, white, or grey. One was a case of "mental blindness," so-called; the other of partial detachment of the retina, followed by reunion.

It is, of course, impossible to discuss the question of colours with persons who, more or less blind thereto from their earliest years, have, by the habit of applying the names they hear employed by others to the several colours of objects around, acquired an apparent perception which rarely fails them, though it is really but an unconscious power of deception.

There are two perfectly distinct conditions or forms of colour blindness, which have their counterparts in the sense of hearing.

Light and sound are alike the product of vibrations, or waves acting on appropriate organs of sense, the medium of light being the hypothetical "ether," and that of sound "matter." The waves of light are infinitely the smaller and more rapid. But it is only within a certain limited range that they can be perceived as light and sound respectively, those above or below these limits being invisible or inaudible, even though, as in the case of the ultra-red or invisible heat rays, they may be otherwise perceived. The red and the violet rays constitute the extremes of the visible spectrum, and those of the musical are approached by the strings of a piano or harp of great compass, the highest of which, if tightened a little more, and the lowest, if slackened, would be rendered silent.

Now, there are some individuals who really cannot hear very high or very low notes perfectly appreciable by others; in rare instances even the whole of the bass has been inaudible to them. But there is a vastly greater number of persons who can hear well enough, but have "bad ears," do not notice wrong notes or slight discords, and could not, to save their lives, distinguish between three or four consecutive notes. To a great extent this is due to neglect, and is capable, in early life at any rate, of much improvement by cultivation or practice; whereas, the first-named defect is obviously irremediable.

*Mutatis mutandis*, these statements apply equally to the sense of colour. Some, indeed not a few, are unable to see the upper or lower portions of the spectrum. Red, for example, is invisible; pure red objects are black, and all mixed pigments into which red enters are darkened thereby. Such a man sees the red lamps of a railway as white lights would appear to others through more or less opaque-smoked glass; and blood to him is like ink; the *μέλαν αἷμα*, of Homer, who was certainly the subject of this defect.

Others, like the unmusical people, see the entire spectrum, but cannot clearly differentiate between its component colours, seeing in it, instead of six (or as some few like Newton himself, seven) distinct colours, only five, four, three, or even two. What they do see no one can exactly say, but Dr. Edridge-Green has thrown much light on the question by a deductive or *a priori* method of experimentation, giving them outline drawings and a box of colours, and tempting them, so to say, by offering just those colours which, according to his theory, they ought to substitute for the proper ones—a trap into which they invariably fall.

The testing of the competency of signalmen, engine-drivers, and the like, should be entrusted only to scientific experts, for intelligent candidates may, by using the correct *names* for colours, innocently impose on others, and we have recently heard, with no small alarm, of men who had been rejected passing after a few weeks spent in learning the conventional designations of colours, the true nature of which they certainly could not apprehend: a most dangerous confusion of names for things.

